# Dietary Habits of Young Poles and Their Selected Determinants: A Review and Implications for Public Health

**DOI:** 10.3390/nu16203561

**Published:** 2024-10-21

**Authors:** Agata Kotowska, Klaudia Sochacka, Rafał Wiśniewski, Sabina Lachowicz-Wiśniewska

**Affiliations:** 1 Institute of Sociology, University of Rzeszów, 35-959 Rzeszow, Poland; akotowska@ur.edu.pl; 2 Department of Medical and Health Science, University of Kalisz (Calisia University), 62-800 Kalisz, Poland; k.sochacka@uniwersytetkaliski.edu.pl; 3 Department of Biotechnology and Food Analysis, Wroclaw University of Economics and Business, 53-345 Wroclaw, Poland; rafal.wisniewski@ue.wroc.pl

**Keywords:** dietary habits, public health, young people, dietary rules, social media, social rules

## Abstract

Background/Objectives: This study investigates the dietary patterns, health behaviors, and related determinants among young people in Poland, amid increasing lifestyle-related health concerns like obesity and poor nutrition. Understanding the factors influencing these behaviors is crucial for crafting effective public health strategies. The objective was to analyze young Poles’ eating habits, their perceptions of health, and the role of various determinants in shaping these behaviors. Methods: A survey was conducted among a representative sample of young Poles, gathering data on eating habits, health perceptions, and lifestyle choices. The survey focused on the respondents’ understanding of healthy eating, sources of nutrition knowledge, and the influence of family, social media, and public health campaigns. Data were analyzed using descriptive statistics, and correlations between health behaviors and socio-demographic factors were examined. Results: The study revealed that 88% of young respondents prioritize health, with 73% acknowledging a link between nutrition and health. While most participants accurately described healthy eating and reported adherence to dietary guidelines, 43% engaged in emotional eating, which often led to compulsive behaviors associated with obesity. Social media, internet articles, and family were primary sources of nutrition knowledge. Despite their knowledge, fruit and vegetable consumption was below recommended levels, and physical activity levels were insufficient in nearly half of the respondents. Only 36% regularly read food labels, with many choosing products containing additives. Appearance-related stress was prevalent among 52% of respondents, while sleep deficits and lack of sufficient physical activity were common. Conclusions: The findings suggest that while young Poles possess a high level of awareness regarding healthy eating, gaps remain in the application of this knowledge, particularly concerning emotional eating and inadequate fruit and vegetable consumption. Public health campaigns should be more effectively targeted to address these gaps and promote sustainable health behaviors from early childhood. Addressing emotional eating, enhancing physical activity, and improving nutrition education through effective channels like social media are key to improving public health outcomes.

## 1. Introduction

Etymologically, the concept of public health pertains to the health of populations as opposed to individual health. It is also associated with actions aimed at prevention and health promotion as part of state health policy. According to the definition adopted by the WHO in the 1940s, “health is a state of complete physical, mental, and social well-being and not merely the absence of disease or infirmity” [1]. This definition can be seen as an ideal type, as fulfilling all of the criteria is either very difficult or impossible. However, it serves as a reference point in the debate on public health and encourages viewing health issues from a population perspective rather than just focusing on individuals. At the same time, it is important to note that these perspectives cannot be completely separated, as the indicator of public health is a state in which individuals’ health problems are solved through community-oriented actions.

Public health issues currently occupy an important place in public discourse. Addressing various problems in this area requires a deep understanding and consistent, targeted systemic intervention. The dynamic changes in recent years, caused by unprecedented global phenomena (e.g., the COVID-19 pandemic), have highlighted existing problems and exacerbated or created new negative phenomena. This situation necessitates the establishment of new frameworks for public health actions and the consideration of a broader perspective. It is essential to include issues such as increasing environmental pollution, deteriorating food quality, the growing share of highly processed food in the global diet, stress, social exclusion, and the need to function under pressure and continuous change, none of which are conducive to maintaining physical and mental health. Therefore, discussions on this topic should consider objective, subjective, and social health measures. Factors determining health include healthy lifestyles and conducive circumstances; a healthy environment; the information, knowledge, and skills necessary to maintain health; proper individual psychosocial development; health-promoting and protective policies; and health services [2].

The increasing focus on public health in the context of identified threats results from observing alarming changes in the population’s condition, as indicated by epidemiological data from various countries. This also concerns undesirable social behaviors that directly impact the population’s health.

Although factors determining health include genetics, the environment, one’s lifestyle (work, study, recreation, diet), and the culture around health, the literature emphasizes that consumers’ lifestyles have the most significant impact on their health, followed by their physical and social environment, genetics, and health care systems [3].

Children and young people are an extremely important segment of society in developing desirable health-promoting attitudes and disease prevention. The future state and vitality of the population, and all related consequences, depend on shaping their knowledge and their awareness of health and a healthy lifestyle. Thompson and Moughan identified three main global trends in nutrition—personalized nutrition, weight control, and food that affect mental health (“mood food”) [4]. This analysis mainly addresses the first two of these issues.

While numerous studies have focused on the importance of a healthy lifestyle and the role of various factors, such as family influence and media, there is still a need for more comprehensive data on the long-term effects of these influences and how they intersect with modern public health challenges. This study contributes to the field by examining young Poles’ eating habits, knowledge, and attitudes, providing valuable insights into the socio-demographic and environmental determinants that shape their behaviors. Identifying key areas of concern, such as the influence of social media and gaps in family-driven health education, the findings contribute to a deeper understanding of the factors impacting public health in younger populations and offer targeted recommendations for intervention strategies.

The study aimed to assess young Poles’ knowledge, attitudes, and eating habits, considering socio-demographic variables (age, gender, lifestyle) and somatic factors (height, body weight). It focused on the nature of eating habits, social determinants, and their potential implications for public health. The study examined young people’s understanding and awareness of various aspects of healthy eating, the values and attitudes they represent, and their sources of knowledge on the topic. It aimed to determine to what extent these habits are shaped by the respondents’ immediate environment, particularly the family as the primary social group, and to what extent media, especially the internet, influences them. Questions were asked about the routine of meal preparation in respondents’ households, the importance parents place on healthy eating, and whether the topic of healthy eating is discussed at home. The study also explored selected behaviors related to nutrition and hygiene and respondents’ satisfaction with their health.

Based on the data obtained, general conclusions were formulated, along with some recommendations for actions related to public health.

## 2. Materials and Methods

The study was conducted in 2024 on a convenience sample of 613 young Poles (Table 1). Participants included students from the University of Kalisz, the University of Rzeszów, the Wroclaw University of Economics, the West Pomeranian University of Technology in Szczecin, and students from various secondary schools: the Ks. Jan Twardowski II High School in Dębica, the Janek Bytnar “Rudy” School of Gastronomy and Hotel Management, and the S. Kopystynski High School of Fine Arts in Wroclaw. The research used a diagnostic survey method with a proprietary electronic questionnaire consisting of 44 closed and semi-closed questions, single and multiple-choice open-ended questions, and a demographic section. A self-administered questionnaire was developed to assess young people’s dietary and social habits. The questionnaire was divided into four sections to gather demographic information and specific dietary habits and knowledge insights. The main categories included the following: demographics (age, gender, height, weight, and place of residence (village, town, or city)); nutritional attitudes (questions regarding the importance of health and the perceived impact of healthy eating); food habits (questions about food choices, such as the frequency of consuming specific food items (e.g., fast food, fruits, vegetables) and the presence of dietary rules); and health perceptions (queries on participants’ satisfaction with their appearance, their experiences with stress, and physical activity) (see Table S1). The questionnaire covered various aspects that influence dietary behavior, including nutritional knowledge, where participants were asked to identify what had most influenced their knowledge of healthy eating, with options including family, friends, media, and educational programs, and food consumption, which assessed the frequency of consumption of various food categories (e.g., fast food, fruits, vegetables, dairy products), ensuring a comprehensive evaluation of the participants’ eating habits. The questionnaire explored how emotions and external factors affect eating behaviors. For instance, participants were asked if their appearance was a source of stress if they engaged in emotional eating during stressful situations, and if they used specific dietary rules in their daily lives. There were also sections addressing the impact of social media, family, and well-known individuals on participants’ dietary choices. This aimed to provide a holistic view of the socio-environmental factors influencing their food intake (see Table S1a–c). The questions were created based on the abbreviated version of the World Health Organization’s Quality of Life Questionnaire (WHOQOL-BREF) and the FFQ6 Food Frequency Questionnaire. The questionnaire was previously validated by estimating its reliability (Cronbach’s alpha = 0.80). The study was conducted in accordance with the Declaration of Helsinki and the ethical document from Calisia University (5/2023). Participants’ personal data were anonymized following the General Data Protection Regulation (GDPR 679/2016) of the European Parliament. Respondents did not provide their names or computer IP addresses. All participants were informed about the study’s objectives and its anonymity prior to giving informed consent to participate and complete the survey. Each questionnaire was submitted to the Google platform, and the final database was downloaded into Microsoft Excel. The questionnaire was anonymous, ensuring the collection of reliable responses.

### Statistic Method

Microsoft Excel 2019 and Statistica 13.3 (StatSoft^®^, Tulsa, OK, USA) were used to process the results statistically. The Shapiro–Wilks W test was used to check the normality of the distribution of variables. The distribution of variables deviated from normal, so non-parametric tests (Mann–Whitney U and Kruskal–Wallis) were used to analyze the data. In each case, a *p*-value of ≤0.05 was taken as the level of significance (α = 0.05).

## 3. Results

### 3.1. The General Characteristics of the Studied Population

The study was conducted on a non-representative group of 613 individuals aged 14–27. Among the respondents, 63% were female and 37% were male. Most participants were from urban areas, with slightly less than half residing in rural areas. The largest group among the respondents were university students, including 41% who were not working and 26% who were employed. High school students accounted for just over one-third of the respondents. The data indicate that the body mass index (BMI) of most respondents was within the normal range (a healthy adult typically has a BMI between 18.5 and 24.9). Additionally, a BMI of <18.5 is classed as underweight, BMI 25.0–29.9—overweight, BMI 30.0–34.9—having grade I obesity, and BMI 35.0–39.9—having grade II obesity.

### 3.2. Health as a Value and the Association of the Concept of “Healthy Eating”

One of the questions posed to young Poles in the initial section of the survey was “How important is health to you?” The results indicate that health is considered a significant life value for the vast majority of respondents (88%). For the remaining respondents, health was of indifferent significance. Simultaneously, there is a well-established belief among young people that healthy eating impacts health—73% believe it has a major effect, while 23% consider it to have a moderate effect (Table S2). This trend is encouraging, as it suggests a high level of awareness about how diet can influence disease prevention or occurrence, thus impacting overall health depending on dietary choices.

When asked “What does healthy eating mean to you?” respondents demonstrated a high level of awareness in their understanding of the concept—only 6 respondents answered, “I don’t know” (1%). They provided more elaborate and concise responses and identified the most important elements of healthy eating. Respondents’ associations revolved around several key issues related to maintaining health. Commonly mentioned elements included the following: (I) balance (balanced diet, meals) (38%), (II) a focus on the quality of food and its health benefits involves consuming vegetables and fruits (18%), (III) eating processed foods and fast foods does not align with the definition of healthy eating (18%), (IV) an awareness that a high intake of simple sugars is detrimental to health (15%), and also (V) the importance of adequate water intake, regular meal consumption throughout the day, monitoring caloric value, and, supplementation (10%).

### 3.3. Sources of Knowledge About Healthy Eating

**Figure 1:**
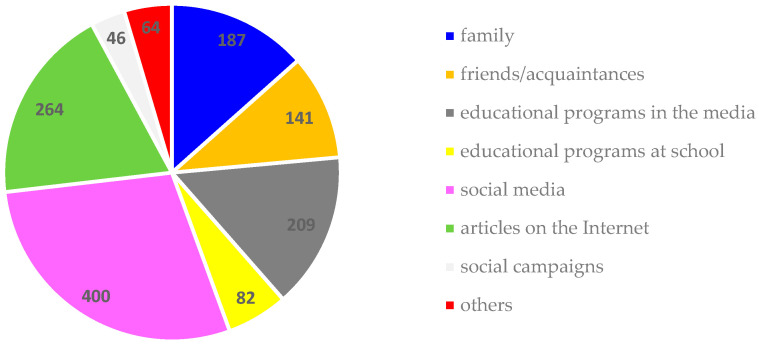
Factors influencing young people’s knowledge about healthy eating.

The primary source of knowledge about healthy eating, as indicated by the respondents, is social media (29%), followed by online articles (19%), educational media programs (such as guides, popular science programs, lifestyle shows, etc.) (15%), and with family ranking fourth (13%). Friends/acquaintances (10%), educational programs in school (e.g., “5 servings of vegetables, fruits, or juice”) (6%), and other sources (5%) follow. The least mentioned source of knowledge was social campaigns (e.g., “Don’t Serve Yourself Illness!” “Don’t Serve Your Child Illness,” “Planning a Long Life”) (3%) (see Figure 1).

When later asked, “Do your parents emphasize healthy eating?” over one-third of respondents answered affirmatively, nearly half chose “sometimes,” and just over one-fifth denied this (see Table S2). Thus, while attention to healthy eating is paid in families of 79% of young people, the intensity varies. Additionally, 81% of respondents reported that meals are regularly cooked at home, 16% said this happens as often as parents and respondents could manage, and 3% reported that no meals are prepared at home. This result indicates that, in most cases, respondents’ families systematically consume home-cooked meals, which requires engagement and suggests an awareness of the benefits of this practice. This is a highly positive trend, given the current lifestyle of most individuals. Concurrently, more than half of the respondents admitted that their parents discussed what constitutes healthy eating with them, whereas 46% did not recall such discussions (see Table S2).

### 3.4. Individual Dietary Habits of the Respondents

The distribution of responses to the question “What does food mean to you?” indicates a tendency to view food not only as a means of maintaining biological functions and survival but also in other terms. Over half (57%) of the respondents indicated that they view food primarily as a means of maintaining basic life functions, while nearly one-third (28%) consider it a way to improve mood, and 15% consider it a form of reward (see Table S2).

To explain these behaviors, 43% of the respondents’ answers suggest the relevance of the phenomenon known as emotional eating. This occurs when eating becomes a response to stress, tension, excitement, or boredom. It serves as a means to alleviate and regulate emotional states such as sadness, anger, loneliness, or anxiety. Rather than satisfying physical hunger, emotional eating addresses “emotional hunger.” The responses of the survey participants may indicate the presence of, or potential for, eating disorders leading to conditions such as obesity (Table S2).

Young individuals generally understand what constitutes healthy eating, but do they apply this knowledge in practice? Most respondents (73%) believe that having fixed meal times is important, whereas 27% disagree. Half of the participants reported eating breakfast daily, while the other half did not (Table 2). Most respondents (62%) have followed a diet or fasting regimen at some point, with 19% doing so for health reasons, 38% for weight loss, 7% due to illness, and 33% for other reasons (Table S2). Simultaneously, 27% of young people pay attention to the calorie content of their meals or snacks, 42% do so occasionally, and 31% do not. Nearly one-third (26%) of the respondents consider how many calories they consume daily, 31% do not, and 43% do so occasionally. Regarding the ingredients of purchased food products, 37% pay attention, 42% do so sometimes, and 21% never. One-third of respondents try to avoid products containing harmful ingredients (preservatives, flavor enhancers, colorants, trans fats, etc.), 43% do so occasionally, and 27% do not check their food in this regard. Only a small percentage of respondents (11%) use apps to check product ingredients while shopping and 15% do so occasionally. A significant proportion of young people (74%) do not use such apps (see Table 2).

Importantly, 63% of respondents indicated that the higher price of healthier products is a barrier to purchasing them, while 37% did not find it significant. Simultaneously, 72% would buy organic products with healthier ingredients if price were not a factor (7% would not purchase them regardless, and one-fifth (21%) were unsure). This trend reflects a broader observation among Poles, who, despite a strong desire to purchase “eco” or “bio” labeled food, are deterred by high prices (see Table 2 and Table S2).

Research indicates that 42% of respondents only occasionally avoid products containing food additives, and 26% do not avoid them at all. Just over one-third (32%) of respondents avoid products with harmful ingredients. A significant proportion (73%) of respondents agreed that fixed meal times are crucial for health, while 27% were not convinced of this correlation (Table 2).

**Figure 2:**
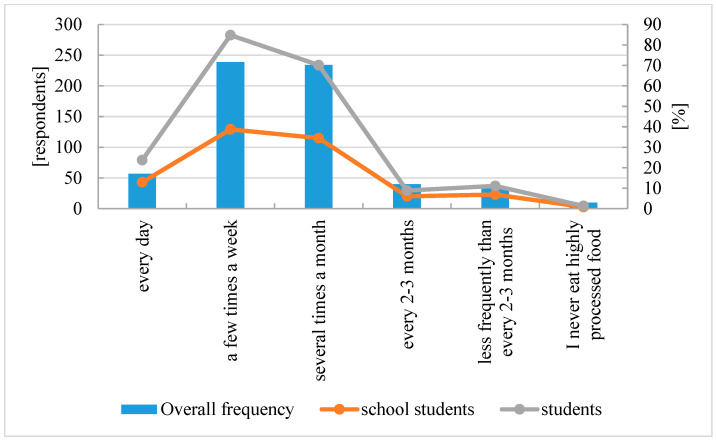
Frequency of consumption of processed foods.

Respondents were also asked about their consumption of highly processed foods (ready meals, semi-finished products, sweet and savory snacks, canned goods, sausages, instant soups and sauces, etc.). Daily consumption of such products was reported by 9% of respondents, 38% consume them several times a week, 38% several times a month, 6% every 2–3 months, 5% less frequently than every 2–3 months, and 4% never consume processed foods. These statistics are concerning, as 76% of individuals consume highly processed foods several times a month or more frequently (see Figure 2).

#### 3.4.1. Personal Dietary Rules

The study inquired about the respondents’ adherence to personal dietary rules, such as eating at specific times, avoiding snacks, limiting sugar intake, avoiding trans fats, refraining from fast food, avoiding energy drinks, and consuming a specified amount of water (see Table 3). Just over half of the respondents (53%) reported having such rules, while 47% had no specific dietary guidelines in their daily routines. When asked about the nature of these rules, responses focused on (I) properly balanced regular meal consumption (32%), (II) controlling caloric intake (28%), (III) including vegetables and fruits in daily meals (18%), (IV) avoiding fast food and processed foods (13%), and (V) no snacking (9%).

However, nearly one-fifth of the respondents (18%) frequently allowed themselves to deviate from their dietary rules, 56% did so occasionally, 20% rarely, and 4% never.

In the conducted study, respondents were also asked if they experienced overeating. One-third (30%) reported doing so frequently, 39% occasionally, and 31% never. Concurrently, 18% of young people confirmed eating more in stressful situations, 27% experience this occasionally, and 55% do not.

#### 3.4.2. Personal Frequency of Eating 

Daily fruit consumption is reported by only 25.9% of respondents; 41.6% consume fruits several times a week, and 12.9% consume them only a few times a month. Regarding vegetables, 38% of respondents stated they consume them daily, 41.4% several times a week, and 6.5% only a few times a month.

Grain products, including cereals, bread, pasta, and flakes, occupy the second tier of the healthy eating and physical activity pyramid. These products are consumed daily by 45.2% of respondents, 35.2% include them in their diet several times a week, and 5.2% several times a month. Dairy products are chosen daily by 40.6% of respondents; 38% consume them several times a week, and 2.4% do not consume these products at all.

Among the respondents, there is a high overall consumption of meat and processed meat products. Daily consumption is reported by 40.8% of respondents, and 43.6% consume these products several times a week. The study did not differentiate between types of meat, such as poultry or red meat.

Current dietary recommendations suggest consuming at least 1–2 servings of fish per week, including one serving of fatty fish. Less than one-third of respondents (26.9%) reported eating fish once a week, and 14.7% consume fish several times a week. In contrast, 23.2% reported consuming fish a few times a month, and 9.1% did not.

Recent studies have suggested that eggs are among the most beneficial foods for human health. Egg consumption among respondents is as follows: 11.3% consume eggs daily, 37.2% several times a week, 25.1% once a week, 16.5% only a few times a month, and 15.3% once every 2–3 months. Additionally, 2.8% of respondents do not consume eggs (see Table 3 and Table 4).

**Figure 3:**
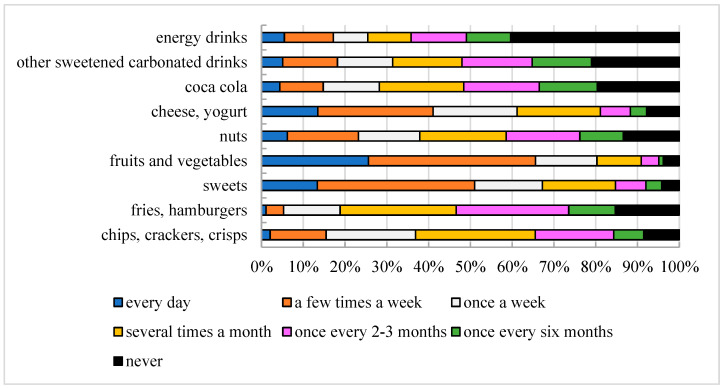
Frequency of consumption of snack groups (*N* = 613); results are presented as a percentage of responses obtained from respondents.

Regarding snack consumption, particularly fast food, the intake among the studied population is not as high as expected, given its characteristics (Figure 3). Less than one-third of respondents (26.9%) reported consuming such food once every 2–3 months, while 27.7% admitted to consuming products from this category several times a month. Only 4.2% of participants opt for fast food several times a week. In contrast, respondents more frequently choose sweets over fast food.

Sugary carbonated beverages are a daily choice for 5% of respondents; 11.7% consume them several times a week, and 27% do not consume these drinks at all. A positive trend can be observed in relation to energy drink consumption. A significant proportion of respondents (40.3%) indicated that they never consume energy drinks, only 5.5% consume them daily, and 11.7% consume them several times a week.

Regarding snacking between meals, 32.8% of respondents admitted to daily snacking, 33.9% snack several times a week, and 12.9% do so once a week. Only 5.7% of respondents said they did not consume additional snacks between main meals. When asked about the types of products they most frequently snack on, the survey results indicate that fruits or vegetables are consumed daily by 25.6% of respondents, 40% snack on them several times a week, and 14.7% do so once a week.

Daily snacking on sweets between meals was reported by 13.4% of respondents, 37.7% do so several times a week, and 16.2% do it once a week. Other products mentioned by respondents as snacks between main meals include chips, crackers, and puffed snacks. Only 2.1% of respondents reported daily consumption of these snacks, 13.4% consume them several times a week, and 21.4% consume them once a week.

Nuts are consumed daily by 6.2% of respondents, 17% consume them once a week between meals, and 20.7% snack on them once a month. Fast food is a snack for 27.7% of respondents, who reported consuming these meals several times a month. A small percentage of respondents, only 1.1%, eat these products every day. Cheese and yogurt are additional products that respondents frequently snack on. Daily consumption of dairy products as snacks is declared by 13.5% of respondents, while 27.6% consume them several times a week, and 20.1% consume them once a week.

Regarding beverages, respondents reported consuming cola between meals. Survey data suggest that 4.4% of respondents drink cola daily between meals, 10.4% consume it several times a week, and 13.4% do so at least once a week. In addition to cola, 5.1% of respondents drink other sugary carbonated beverages daily, 13.1% consume them several times a week, and 13.2% drink them once a week. A proportion of 5.5% of respondents consume energy drinks daily between meals, 11.7% consider them a form of snacking several times a week, and 8.2% consume them once a week.

#### 3.4.3. Selected Meal Categories

Nearly half (49.1%) of respondents said they eat breakfast daily, while 27.2% remember having their first meal only a few times a week. On the other hand, 9.1% of the respondents consume this meal once a week, and 5.7% completely omit it from their diet. As for the consumption of second breakfasts, the results are as follows: 25.1% of the respondents eat a second breakfast daily, 30.6% a few times a week, and 9.8% only once a week. Nearly one-fifth (18.9%) of respondents admitted to not preparing second breakfasts.

**Figure 4:**
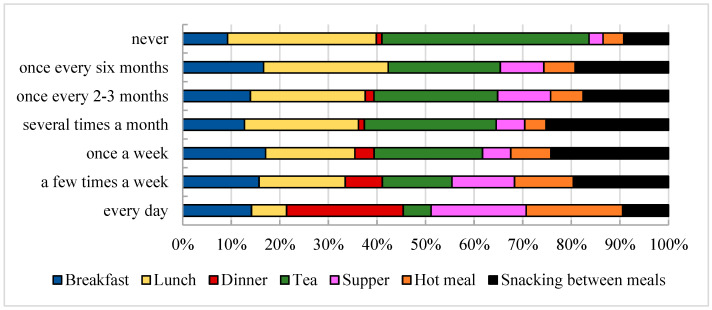
Frequency of consumption of meals groups (*N* = 613); results are presented as a percentage of responses obtained from respondents.

The situation is entirely different regarding lunch consumption among young people. Data indicate that 83.2% of respondents have lunch daily, 13.2% several times a week, and only 2.1% of individuals eat lunch once weekly (see Figure 4). A proportion of 19.9% of respondents consume an afternoon snack daily, with another 24.8% preparing this type of meal several times a week, while 26.3% do not include an afternoon snack in their diet. After lunch, dinner is the most important meal for the surveyed individuals—67.9% of them eat it daily and 22.3% of them report eating it a few times a week. The data show that an important issue is consuming a hot meal. Most respondents (68.7%) said they eat such a meal every day, 20.9% a few times a week, and 4.4% at least once a week (see Figure 4).

### 3.5. Appearance and Hygiene of Life

In the study, 52% of respondents said their appearance is a source of stress (48% responded negatively). A quarter of respondents (25%) are satisfied with their appearance, 23% are dissatisfied, and 51% are somewhat satisfied but would like to change certain aspects of their appearance. At the same time, 51% stated that their weight is within the normal range, 15% declared being underweight, 13% weigh up to 5 kg more than their desired weight, 11% weigh 5 to 10 kg more, and 7% weigh more than 10 kg over their desired weight.

Most respondents are satisfied with their health, while the remaining 38% disagree. As noted above, health status is largely a result of lifestyle, with nutrition and physical activity being the most significant factors. Daily physical activity is reported by 14% of respondents; 33% are active several times a week, 18% engage in physical activity a few times a month, 24% declare occasional physical activity, and 9% are not physically active. Therefore, the data indicate that 33% of young people have little or no physical activity.

**Figure 5:**
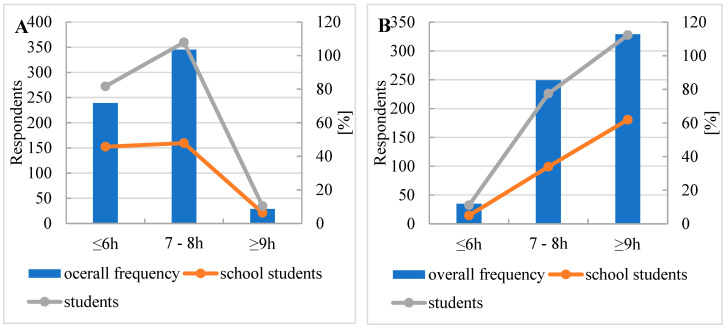
Number of hours of sleep (**A**) on weekdays and (**B**) on weekends.

When asked about weekday sleep duration, respondents replied that 38% sleep 6 h or less, 56% sleep 7–8 h, and 4% sleep 9 h or more (See Figure 5). On weekends, only 5% of respondents sleep 6 h or less, 40% sleep 7–8 h, and 53% sleep 9 h or more.

## 4. Discussion

Individual opinions on health as a value and the concept of “healthy eating” may stem from practical life considerations and the influence of immediate surroundings on attitude formation and cultural content. Contemporary culture, especially its iconosphere, emphasizes health, vitality, and beauty, with a strong anti-aging message (e.g., anti-aging medicine, age-correct cosmetics, wellness, etc.) that effectively promotes and sustains certain attitudes in this domain. Dietary habits are a primary expression of concern for maintaining health and physical condition, aligning with current beauty standards. They may also lead to social exclusion, a reality that young people know. On the one hand, young consumers are a significant target group for producers of highly processed foods (snacks, ready meals, sugary drinks, energy drinks, etc.), and on the other hand, for companies offering products to clients with specific and high demands related to diet (e.g., specialized diets, vegetarianism, veganism, etc.). Notably, healthy eating has become an essential aspect of consumer identity, especially among affluent and better-educated individuals, with chosen dietary practices contributing to individual or collective identity [5].

The data obtained show considerable alignment with related issues, such as the results from the European survey “Emotional Health of Generation Z and Millennials: What Concerns Young Europeans?” conducted in June 2023 by the pharmaceutical company Merck and regarding young Europeans from Generation Z (19–25 years) and millennials (26–36 years). The study was conducted in 12 countries and with 7495 respondents, of whom 641 were from Poland [6]. It covered aspects of emotional and physical health. Young Poles indicated that the most critical elements of their lives are personal relationships (95%), emotional health (95%), and physical health (93%). Concurrently, according to respondents, the most essential habits for ensuring health included adequate sleep and rest (79.2%), regular exercise and physical activity (76.5%), and healthy eating habits and proper diet (66.1%) [7].

Our research also highlights the importance of adequate water intake, regular meal consumption throughout the day, the monitoring of caloric value, and, less frequently, supplementation. According to the EAT report [8], a rational approach to diet, primarily based on plant-based products, positively impacts health and the environment. Consuming minimal red meat, limiting highly processed products, and favoring large quantities of vegetables and fruits, whole grains, legumes, seeds, and nuts will beneficially improve health [8].

The results of our study highlight the significant role that the internet, particularly social media, plays in providing knowledge, shaping needs, and influencing behavioral attitudes among young people. Studies conducted over the years illustrate that nearly one in four residents of the European Union searches for information about broadly defined health topics online [9].

Following social media, family is the next significant factor in shaping knowledge. Research also indicates a tertiary role for other small social groups (friends, acquaintances) in this context. An important conclusion is the apparent ineffectiveness of preventive and educational social campaigns, which may stem from the need for television messages to appeal more to the younger generation that is immersed in the digital world and the unengaging format of these campaigns.

The positioning, at fourth place, of family as a source of knowledge about healthy eating leads to much speculation. It might reflect, among other factors, a loosening of family bonds, a diminishing role of parents in children’s lives, limited or specific communication with children (e.g., restricted coding instead of more developed discourse) [10], or a lack of interest in healthy eating within the respondents’ households (though the collected data do not confirm this). This may also suggest that young people do not consider implicitly transferred knowledge—derived from observing and participating in daily family routines, lifestyle habits, and parental attitudes toward nutrition. Observation, imitation, and participation in daily practices are fundamental elements of socialization and natural ways of conveying information and enriching knowledge. Young people might not recognize the family’s role in shaping their worldview and behaviors. When later asked “Do your parents emphasize healthy eating?” over one third of respondents (32%) answered affirmatively, nearly half (47%) chose “sometimes,” and just over one fifth (21%) denied this. Thus, while attention to healthy eating is paid in families of 79% of young people, the intensity varies. Additionally, 81% of respondents reported that meals are regularly cooked at home, 16% said this happens as often as parents and respondents can, and 3% reported that no meals are prepared. This result indicates that, in most cases, respondents’ families systematically consume home-cooked meals, which requires engagement and suggests an awareness of the benefits of this practice. This is a highly positive trend, given the current lifestyle of most individuals. Concurrently, more than half of the respondents (54%) admitted that their parents discussed what constitutes healthy eating with them, whereas 46% did not recall such discussions.

It is also important to note that overweight and obesity in children and adolescents, which currently represent a growing global issue, primarily result from poor dietary habits, excessive energy intake in daily food, and decreasing physical activity [11]. According to a WHO report, overweight and obesity were observed in 32% of Polish children aged 7–9 years, ranking 8th among surveyed countries in Europe [5,12]. The role of parents in establishing healthy dietary habits is substantial. Incorrect dietary patterns transmitted in early childhood hinder optimal development in subsequent years and predispose individuals to various diseases, including obesity. Many specialists note a reciprocal correlation between nutritional status in early childhood and health in adulthood [13]. Researchers argue that, although genetic factors play a significant role in obesity, dietary habits influenced by family practices and physical activity are of utmost importance. Numerous studies suggest that shared meal consumption reduces the risk of obesity and that eating meals with parents at home fosters better dietary habits [14]. Therefore, promoting health-oriented behaviors from the early stages of socialization is essential to significantly impact health later in life and prevent the development of various diseases. A rational diet and ensuring appropriate energy levels during early childhood are protective factors against overweight or obesity, commonly observed among Polish children and adolescents [13]. Consequently, experts emphasize the ongoing need to educate parents to take appropriate actions to influence their children’s health by providing suitable nutritional conditions [15].

The definition provided in Article 2 of Regulation (EC) No 178/2002 of the European Parliament and Council of 28 January 2002, [16] states that “food (or foodstuff) means any substance or product, whether processed, partially processed, or unprocessed, intended for human consumption, or which can reasonably be expected to be consumed by humans. It includes drinks, chewing gum, and any substance, including water, deliberately added to food during its production, preparation, or processing. Food consumption aims to provide the human body with essential nutrients (building, energy, and regulatory)” [16].

The distribution of responses to the question “What does food mean to you?” indicates a tendency to view food not only as a means of maintaining biological functions and survival but also in other terms. Over half (57%) of the respondents indicated that they view food primarily as a means of maintaining essential life functions, while nearly one-third (28%) consider it a way to improve mood, and 15% consider it a form of reward. Food consumption often addresses psychological needs, such as serving as a reward or punishment, providing comfort, expressing and experiencing emotions, substituting for relationships, gaining control, and reducing anxiety and tension. Chanduszko-Salska’s [17] study suggests that high levels of perceived stress contribute to a focus on food and to coping with tension through eating. This perception of food can, consequently, lead to adverse health outcomes, including overweight and obesity [18].

To explain these behaviors, 43% of the respondents’ answers suggest the relevance of the phenomenon known as emotional eating. This occurs when eating becomes a response to stress, tension, excitement, or boredom. It serves as a means by which to alleviate and regulate emotional states such as sadness, anger, loneliness, or anxiety. Rather than satisfying physical hunger, emotional eating addresses “emotional hunger.” The responses of the survey participants may indicate the presence of, or potential for, eating disorders leading to conditions such as obesity.

Individuals particularly susceptible to emotional eating include those with a high need for control and self-awareness, neurotic individuals, those who are highly reactive to emotional stimuli, and those with low self-esteem and a distorted self-image. The results suggest that determining the underlying behaviors of respondents would require further in-depth research.

In recent years, there has been increased attention to healthy eating, with a growing trend towards natural products that do not contain sugar, flavor enhancers, or preservatives. Conversely, highly processed products with long ingredient lists, artificial flavors, colorants, and extended shelf lives still appear on store shelves. These products are often chosen for their stronger flavors or lower prices. There remains a need to raise public awareness and promote safe dietary choices. Only sustained and consistent efforts highlighting the health benefits of a well-balanced diet can significantly influence consumer choices on a larger scale. Socioeconomic factors also play a crucial role. In our study 60% of respondents indicated that the higher price of healthier products is a barrier to purchasing them, including 72% who would buy organic products with healthier ingredients if price were not a factor. This trend reflects a broader observation among Poles, who, despite a strong desire to purchase “eco” or “bio” labeled food, are deterred by high prices. According to IQS research [19], 62% of respondents wish to buy such products but often refrain from paying the costs. Most respondents (68%) considered these products too expensive [20].

Food additives can be categorized into flavor enhancers, preservatives, colorants, and emulsifiers. Not all of these are harmful. Moreover, the WHO/FAO Expert Committee on Food Additives has established the concept of acceptable daily intake (ADI), which defines the maximum amount of a substance (per kilogram of body weight per day) consumed without posing a health risk. Nonetheless, high consumption of processed foods can accumulate these substances in the body, potentially resulting in adverse health effects [21]. Processed products, with their flavor enhancers, appeal to children, adolescents, and adults, especially when time constraints limit meal preparation. Research indicates that 42% of respondents only occasionally avoid products containing food additives, and 26% do not avoid them at all. Just over one-third (32%) of respondents avoid products with harmful ingredients.

Regular meal consumption is a key dietary recommendation. While regular eating can vary for individuals, it fundamentally involves consuming a specific number of meals at relatively consistent intervals. Some people may find five meals a day effective, while others may opt for three or four. Eating meals systematically and avoiding frequent snacking between main meals is essential. An appropriate meal schedule supports proper metabolism and blood glucose levels. Furthermore, a consistent meal pattern prevents the consumption of salty and sweet snacks and ensures better concentration through a steady energy supply [18]. A proportion of 73% of respondents agreed that fixed meal times are crucial for health, while 27% were not convinced of this correlation.

As previously mentioned, the role of food extends beyond merely filling the stomach and satisfying physiological hunger. It also involves cultural elements and social functioning, strengthening interpersonal bonds. Eating is associated with the experience of various emotions. This connection is not without reason. The stomach is a highly innervated organ. Stressful situations, chronic tension, and emotional disturbances can disrupt the nervous system, potentially leading to gastrointestinal issues. Eating is an integral part of our lives. However, the statistics are concerning, especially in developed countries, where eating disorders, primarily characterized by uncontrolled overeating, are a significant issue. Overeating goes beyond the physiological need for hunger satisfaction and becomes a means of psychological and emotional fulfillment, leading to various other health consequences [22].

Recent research has unequivocally demonstrated that vegetables and fruits should form the cornerstone of our diet. They provide numerous essential nutrients (minerals, vitamins, polyphenols) and significantly reduce the incidence and mortality of cardiovascular diseases, diabetes, and cancer, as well as extend the human lifespan [23]. However, the results obtained among young people indicate a continued need for education because daily fruit consumption was reported by only 25.9% of respondents; regarding vegetables, 38% of respondents stated they consume them daily.

Grain products, including various cereals, bread, pasta, and flakes, occupy the second tier of the healthy eating and physical activity pyramid; in turn, these products were consumed daily by 45.2% of respondents. In turn, dairy consumption is particularly important due to its calcium content, which was chosen daily by 40.6% of respondents. This mineral is crucial for bone and dental health and performs various regulatory functions in the body. The typical calcium intake in Poland covers approximately 60% of the daily requirement, with milk and dairy products being the primary dietary sources. For example, 2% milk contains 118 mg of calcium per 100 g, while parmesan cheese contains 1380 mg per 100 g. Notably, calcium from dairy products is the most bioavailable [24,25].

Among the respondents, there is a high overall consumption of meat and processed meat products. The study did not differentiate between types of meat, such as poultry or red meat. Several studies have highlighted the detrimental effects of consuming red meat due to its high trans fat content, which can contribute to various diseases, particularly metabolic disorders such as obesity, hypertension, and type 2 diabetes. Cardiovascular diseases are the leading cause of death in Poland, accounting for 39.4% of all deaths in 2019. Their prevalence is significantly influenced by lifestyle factors (lack of physical activity, excessive stress) and poor diet, particularly high consumption of meat and animal fats [26,27].

According to the latest Institute of Agricultural and Food Economics report, fish consumption in Poland is increasing yearly. In 2021, the per capita consumption was 14 kg (globally 20.5 kg). Poles increasingly choose marine fish, such as herring, mackerel, and pollock [28]. Fish are a significant source of polyunsaturated fatty acids (omega-3 and omega-6) known for their anti-inflammatory properties. Including fish in one’s diet, particularly fatty marine fish, is beneficial due to their high omega-3 and iodine content [28,29]. The anti-inflammatory effects of fish positively impact the nervous system and contribute to the prevention of cardiovascular diseases while also positively affecting the immune system due to vitamin D3 content [30,31]. Current dietary recommendations suggest consuming at least 1–2 servings of fish per week, including one serving of fatty fish. However, less than one-third of respondents (26.9%) reported eating fish once a week; in contrast, 9.1% do not consume fish.

Recent studies suggest that eggs are among the most beneficial foods for human health (Table 2). Current research indicates that there is no need to avoid eggs due to their high cholesterol content, as previously thought, because saturated fatty acids, rather than cholesterol, are the primary contributors to an increased risk of cardiovascular diseases. Eggs are a valuable food source, providing protein with optimal proportions of essential and non-essential amino acids to support the body’s structural functions. Eggs are also an excellent source of lutein, which has beneficial effects on vision [25,32]; however, just 11.3% of respondents reported consuming eggs daily, and 37.2% several times a week (Table 2).

According to the World Health Organization (WHO), sugar intake should not exceed 10% of one’s daily energy intake. Added sugars increase the risk of obesity and diabetes and negatively affect other cardiovascular risk factors. A significant proportion (37.7%) consume sugary snacks several times a week, and 13.4% consume them daily. Similarly, salty snacks are consumed several times a week by 13.4% of respondents, with 28.7% consuming them several times a month and 21.4% consuming them once a week. The WHO also warns that the safe level of salt consumption is a maximum of 5 g per day. Excessive salt intake, which can easily be exacerbated by additional salty snacks consumed throughout the day, contributes to conditions such as hypertension and other cardiovascular diseases. Therefore, it is crucial to minimize salt additives in our diet.

It is noteworthy that a meta-analysis of 11 prospective studies involving 310,819 individuals found that high consumption of sugary drinks (1–2 servings per day) compared with low consumption (<1 serving per month) was associated with a 26% increased risk of diabetes and a 20% increased risk of metabolic syndrome [33]. A prospective study conducted by Larsson et al. [34] suggests that high consumption of sweetened beverages, including non-alcoholic ones, significantly contributes to an increased risk of stroke. For individuals consuming more than two servings of sweetened beverages daily, the risk of stroke increased by 22% compared with those who consumed two servings per week [34,35].

The belief that breakfast is the most important meal of the day is one of the most well-established statements in dietetics. Numerous studies suggest that breakfast improves concentration, memory, and practical daily functioning. Second breakfast refers to an additional meal consumed mid-morning. This practice, and having more than three meals daily, can help maintain energy levels and prevent hunger between main meals, promoting better overall health and cognitive function. Previous studies indicate that children who skipped second breakfasts were significantly more likely to report symptoms such as headaches, mood declines, irritability, and problems with peers. Moreover, they were observed to have greater difficulties with learning. In contrast, a positive correlation has been observed among those who consume breakfast regarding overall health and well-being. These individuals are characterized by a lower risk of developing hypertension, metabolic syndrome, diabetes, and obesity [36].

For years, one of the recommendations concerning the principles of healthy eating has been to consume 4–5 meals a day at regular intervals [25]. However, this approach has slightly changed in recent years, and consuming only three meals tailored to the daily rhythm is now acceptable. Nutrition specialists increasingly emphasize a rational approach to diet rather than strictly adhering to rigid rules and regulations. It is advisable to approach meal planning individually. A study conducted among 219 volunteers who have type 2 diabetes showed that the first group, which was given two substantial meals a day (breakfast and lunch), experienced greater weight loss, better glycemic control, and less liver fat accumulation compared with the same parameters of individuals in the second group, who consumed six meals throughout the day [35]. However, based on their practice, most dietitians suggest incorporating five meals into the daily diet, consumed every 3–4 h.

In 2022, a study on stress in daily life was conducted among the Polish population. For the majority of respondents (78%), the concept of “stress” was associated with experiencing psychological discomfort [37]. Stress can be triggered by various stressors (physical, psychological, social), and resilience to stress depends on the individual characteristics of the organism. These include internal human strengths and the ability to develop effective coping strategies in difficult situations. Children and adolescents, due to their underdeveloped defense mechanisms, are particularly vulnerable to the negative effects of stress. One reason for stress among young people is their physical appearance, which is seen as one of the leading derivatives of dietary habits, especially in body weight. In turn, in our study, as many as 52% of respondents said their appearance is a source of stress (48% responded negatively).

The results indicate that only one-quarter of young people are unconditionally satisfied with their appearance, meaning they accept it and do not wish to change anything about it. Slightly fewer expressed the opposite view, while more than half have an ambivalent attitude toward this issue. Ambiguity in self-assessment of appearance can be a source of frustration, leading to lower self-esteem and reduced social activity. In some cases, it can also be the basis for developing body dysmorphic disorder and, in cases of concerns regarding body weight, eating disorders. An additional concern is that their appearance is stressful for most respondents. This highlights the scale of the cultural (media and peer) pressure that youth feel regarding their appearance. This pressure is often driven by idealized images of individuals on social media, who serve as benchmarks for appearance and behavior among young people. Online content is particularly influential for adolescents, as confirmed by the results of this research. In the study of Grabe et al. [38], a meta-analysis of experimental and correlational studies was performed to assess the media’s impact on women’s body image concerns. This study demonstrated a strong link between exposure to idealized images in the media and body image issues, as well as eating disorders, among women [38].

As noted in the study, health status is largely a result of lifestyle, with nutrition and physical activity being the most significant factors. The data indicate that 33% of young people have little or no physical activity. This represents a significant percentage, considering the increasing health risks associated with the current lifestyle, where many activities are performed in a sedentary manner or in conditions of very limited movement (education, work, leisure activities). Considering the widespread availability and consumption of cheap, addictive, and low-quality food, this characterizes an environment that is highly detrimental to maintaining health. In the context of public health, it is important to emphasize the urgent need for intervention to promote health-positive social behaviors, as current statistics in many aspects already show very negative trends that will continue to worsen [39].

An important factor influencing health (and indirectly appearance) is sleep. This applies to people of all ages, and research has shown that sleep disorders, including insufficient sleep duration and circadian rhythm disturbances, increase the risk of cardiometabolic diseases. Greater sleep irregularity was also correlated with a higher 10-year risk of cardiovascular diseases, increased obesity, hypertension, fasting glucose levels, hemoglobin A1C levels, and diabetes status. Additionally, greater sleep irregularity was associated with increased perceived stress and depression, psychiatric factors integrally linked with cardiometabolic diseases [40]. Evidence shows that sleep regulates processes that are important in regulating endocrine functions that are involved in tissue regeneration and remodeling [41]. Due to rapid cognitive development, adolescence is a critical period in which targeted preventive actions, including the inculcation of healthy sleep habits, can have a long-term impact on health [42]. Modern adolescents are exposed to unusually high light during the day. Long after sunset, they are exposed to artificial, bright light from many sources, including mobile phone use. It is worth noting that early or middle adolescence may be associated with increased sensitivity to light. It is, therefore, assumed that people with irregular sleep patterns experience greater variability in light exposure times, disrupting the circadian rhythm and resulting in adverse health effects. Sleep regularity (consistent sleep and wake times) determines sleep quality and is closely related to overall health [42].

Moreover, the data indicate that a significant portion of young people (38%) experience significant sleep deficits during the week, which can negatively affect their daily functioning (e.g., cognitive functions) and their future health. It is also worth noting the connection between sleep and stress. Effective and adequate long sleep promotes relaxation, while stress increases susceptibility to sleep disorders and is strongly associated with insomnia. The results suggest that respondents who sleep too little during the week try to catch up on sleep during the weekend. Additionally, other respondents devote a significant amount of time to sleep during the weekend.

Thus, the study stands out as one of the few recent research efforts focusing specifically on the post-pandemic dietary habits of young Poles. This context is particularly relevant as the COVID-19 pandemic significantly altered lifestyles, stress levels, and food availability, which may have had long-term impacts on eating behaviors. The survey conducted during this period captures young people’s evolving dietary habits and health awareness in a critical phase of social transformation, making it an important study within the current research landscape.

The novelty of this study concerns emotional eating and its underexplored implications and public health challenges. The article brings new insight into the phenomenon of emotional eating among Polish youth. Although emotional eating has been studied globally, this is one of the few studies that specifically target its prevalence among young people in Poland, particularly in relation to stress and appearance-related pressures. This is a novel direction within the context of public health in Poland, as it links mental health and eating behaviors more explicitly than previous studies. Furthermore, the study highlights how ineffective public health campaigns are in reaching young people, despite their awareness of the importance of healthy eating. This observation calls for a re-evaluation of public health strategies and is a novel contribution that could influence future public health policies targeting younger populations, especially with regard to social media engagement.

In the context of public health implications—the study provides actionable insights into the gaps between nutritional knowledge and actual eating behaviors. It underlines the discrepancy between young people’s awareness of healthy eating and their engagement in harmful behaviors, such as frequent processed foods and emotional eating. This makes the research valuable for future interventions and education programs aimed at closing this gap. In turn, in the context of family and social media influences—the findings emphasize the significant role that family and social media play in shaping the dietary habits of young people. This adds to the current understanding of dietary influencers and raises critical questions about the effectiveness of family influence compared with the overpowering role of digital media in shaping nutritional behaviors.

## 5. Conclusions

The study reveals that young people in Poland prioritize health, with 88% considering it important and 73% recognizing the connection between health and diet. Despite good awareness of healthy eating, 43% exhibit emotional eating behaviors, which can lead to weight issues. Public health campaigns need to reach this group more effectively, with most relying on social media and family for health information. Gaps exist in applying healthy habits, especially in stress management, fruit and vegetable consumption, and physical activity. The study suggests that public health efforts should focus on emotional regulation, access to healthy food, and the promotion of physical activity from an early age. 

## Figures and Tables

**Table 1:** The general characteristics of the studied population.

Sociodemographic Characteristics		*N* = 613	[%]
Gender	Female	384	63
	Male	229	37
Age	14–18	188	31
	19–26	386	63
	≥27	39	6
Place of residence	Rural area	280	46
	City	333	54
Occupational situation *	Student in high school	201	33
	Student (not working)	250	41
	Student (working)	162	26
BMI (kg/m^2^)	<18.5	92	15
	18.5–24.9	402	66
	25.0–29.9	86	14
	30.0–34.9	25	4
	≥35.0	8	1

* Explanations: A working student is an individual enrolled in an educational program, typically at a university or college, while holding a part-time or full-time job; this student balances academic responsibilities with employment to support their education, gain experience, or meet financial needs. A non-working student, on the other hand, is a student who is solely focused on their studies and is not engaged in any form of paid employment during the academic year; their time is primarily dedicated to academic pursuits without the additional job responsibilities.

**Table 2:** Selected views and dietary habits of the respondents (*N* = 613).

Respondents’ Views and Habits			
Survey Questions:	Yes	No	Sometimes
Do you agree with the statement that having regular meal times is important?	450	163	
Do you eat breakfast every day?	303	310	
Have you ever followed a diet or fasting regimen?	384	229	
Do you pay attention to the calorie content of your meals and snacks?	163	194	256
Do you consider how many calories you have consumed throughout the day?	168	255	190
Do you pay attention to the ingredients of the food products you purchase?	225	127	261
Do you try to avoid products containing harmful ingredients (e.g., preservatives, flavor enhancers, colorants, trans fats, etc.)?	190	168	255
Do you use apps to check product ingredients while shopping (e.g., Zdrowe Zakupy/Health Shopping, Wiesz Co Jesz/Know What You Eat)?	72	451	90

**Table 3:** Frequency of selected food group consumption according to respondents’ age (*N* = 613).

The Frequency of Consumption	Every Day			A Few Times a Week			Once a Week			Several Times a Month			Once every 2–3 Months			Once every Six Months			Never			p *
	Me	Q1	Q3	Me	Q1	Q3	Me	Q1	Q3	Me	Q1	Q3	Me	Q1	Q3	Me	Q1	Q3	Me	Q1	Q3	
Fast food	17	14	29	20	18	20	19	17	22	20	18	21	20	17	22	17	16	24	21	19	24	0.326
Sweet snacks	19 b	15	20	19 a	16	21	20 a	18	22	20 a	18	22	20 a	19	22	23 a	20	25	23.5 a	22	42	<0.001
Sweets	20	18	22	20	17	22	20	17	22	20	19	22	20	19	23	18	17	23	21	20	24.5	0.103
Sweetened carbonated drinks	20 a†	17	22	19 a	16	21	19 b	17	22	19 b	16	21	20 a	17.5	21.5	20 b	19	22	21 b	19	23	<0.001
Energy drinks	19	18	21	19	18	21	20	17	22	20	17	22	20	18	21	20	18	23	20	18	22	0.369
Fruit	20	17	23	20	18	22	20	17	22	20	18	22	19	18	21	18	18	19	17	17	20	0.668
Vegetables	20	18	22	19	18	21	20	18	22	19	18	21	17	15	21	20	19	20	19.5	16.5	31.5	0.117
Grain products	20	17	22	20	17	22	20	18	22	20	19	22	20	18	26	19	17	20	19.5	16	22	0.947
Meat and cold cuts	20	17	22	20	19	22	20	19	21	20	19	22	20.5	18.5	21	18.5	17.5	24	22	18.5	24	0.133
Milk and milk products	20	17	22	20	18	22	20	17	22	21	20	22	19	17	27	17.5	14	21	20	19	22	0.213
Fish	20	18	32	20	18	23	20	17	22	20	18	22	20	18	21	19	17	20	19	17	22	0.082
Eggs	20	17	23	20	18	22	20	18	22	20	17	21	20	19	22	19	18	20	19	17	21	0.266

†a–b, the statistically significant difference in age value depending on the change in frequency of consumption of selected food groups. * Kruskal–Wallis test. Me = median; Q1 and Q3 = first and third quartiles.

**Table 4:** Frequency of selected food group consumption according to respondents’ BMI (*N* = 613).

The Frequency of Consumption	Every Day			A Few Times a Week			Once a Week			Several Times a Month			Once every 2–3 Months			Once every Six Months			Never			p *
	Me	Q1	Q3	Me	Q1	Q3	Me	Q1	Q3	Me	Q1	Q3	Me	Q1	Q3	Me	Q1	Q3	Me	Q1	Q3	
Fast food	24.2	19.6	43.4	21.4	19.0	24.8	21.9	19.7	24.1	21.3	19.3	24.5	21.4	19.5	24.3	21.6	19.6	23.9	20.3	19.1	20.8	0.338
Sweet snacks	21.7	18.7	23.6	21.0	19.0	24.7	21.4	19.4	24.4	21.4	19.7	24.2	22.6	20.5	26.0	21.3	19.1	23.4	20.9	19.3	21.5	0.404
Sweets	21.0	19.5	25.2	21.0	19.0	23.6	21.7	19.4	24.7	21.8	20.3	24.5	23.4	20.6	25.2	20.6	19.6	22.8	22.5	20.3	25.7	0.103
Sweetened carbonated drinks	21.3 c†	19.6 e	26.3 d	20.8 e	19.0 e	24.6 e	21.4 e	19.4 d	24.5 e	21.5 c	19.4 c	23.6 c	22.6 c	19.4 d	24.7 d	22.0 c	20.5 c	23.4 d	21.2 c	19.3 c	23.9 c	0.023
Energy drinks	21.8	18.9	27.7	22.3	18.9	24.8	22.7	20.2	25.2	21.7	20.1	24.2	21.5	19.7	23.6	21.2	19.6	23.6	21.0	19.1	24.1	0.286
Fruit	21.1	19.4	22.4	21.5	19.5	24.4	21.8	19.4	24.8	24.5	19.4	24.8	20.7	18.4	22.6	20.8	20.3	21.4	24.2	21.9	28.9	0.306
Vegetables	21.2	19.5	23.8	21.8	19.5	24.7	21.3	18.9	24.7	21.5	19.8	24.7	21.3	19.0	22.6	20.8	20.3	22.3	20.3	16.4	31.1	0.572
Grain products	21.2	19.4	24.2	21.9	19.4	24.8	21.5	18.9	24.2	21.3	19.8	23.7	21.5	20.1	24.2	20.3	18.8	21.1	21.0	18.6	28.6	0.171
Meat and cold cuts	21.9	19.7	25.1	21.4	19.3	24.1	21.1	19.1	24.5	20.5	18.9	22.1	21.1	18.1	22.3	19.9	18.3	21.5	20.8	18.2	21.9	0.497
Milk and milk products	21.8	19.7	25.1	21.6	19.5	24.2	20.8	18.6	24.1	20.5	17.8	22.4	21.5	17.9	24.3	22.1	20.3	22.0	21.1	20.2	22.6	0.115
Fish	22.9 d	20.6 c	28.7 c	21.4 d	20.2 c	25.1 c	21.8 c	19.2 e	24.6 d	20.8 d	18.9 d	23.1 d	22.5 d	20.2 c	24.6 e	21.4 d	20.1 d	24.4 c	20.5 d	17.9 d	22.7 d	0.003
Eggs	22.4 e	20.0 d	24.9 e	21.5 c	19.6 d	24.7 d	21.7 d	19.6 c	24.7 c	20.8 d	18.8 e	23.6 c	21.6 e	19.0 e	24.8 c	20.1 e	18.4 e	20.8 e	19.7 e	17.6 e	21.1 e	0.001

†c–e, the statistically significant difference in BMI depending on the change in frequency of consumption of selected food groups. * Kruskal–Wallis test. BMI = body mass index; Me = median; Q1 and Q3 = first and third quartiles.

## Data Availability

The original data presented in this study are available at the following link: https://forms.office.com/Pages/DesignPageV2.aspx?subpage=design&token=eb15a7f34fe7492bb6c92726bcab68c5&id=HcphhVDqfUCnABtQYrPB3_qG-tFly3TBUMjNXSE9SN0UyMU9GTkwxN1JWVjlOVTZaWS4u&tab=0&analysis=true (access on 30 October 2024).

## Supplementary Materials

The following supporting information can be downloaded at https://www.mdpi.com/article/10.3390/nu16203561/s1. Table S1: Questionnaire. Table S1a: How often do you eat the following foods? Table S1b: How often do you have meals? Table S1c: Which products do you snack on between meals, and how often? Table S2: Questions from the form.
