# Supplementary material for: Dietary Habits of Young Poles and Their Selected Determinants: A Review and Implications for Public Health
Source: Nutrients. 2024 Oct 21;16(20):3561. doi: 10.3390/nu16203561 (PMC11510485; doi:10.3390/nu16203561)
Supplement: Supplementary file 1 [file nutrients-16-03561-s001.zip › nutrients-3219906-supplementary.pdf]

Type of the Paper (Article, etc.)

Dietary Habits of Young Poles and Their Selected Determinants: A Review and Implications for Public Health

Agata Kotowska[1], Klaudia Sochacka[2], Rafał Wiśniewski[3] and Sabina Lachowicz-Wiśniewska[2,3],*

[1] Institute of Sociology, University of Rzeszów; akotowska@ur.edu.pl;
[2] Department of Medical and Health Science, University of Kalisz (Calisia University); k.sochacka@uniwersytetkaliski.edu.pl, s.lachowicz-wisniewska@uniwersytetkaliski.edu.pl
[3] Department of Biotechnology and Food Analysis, Wroclaw University of Economics and Business, 53-345 Wroclaw, Poland; sabina.lachowicz-wisniewska@ue.wroc.pl, rafal.wisniewski@ue.wroc.pl
* Correspondence: s.lachowicz-wisniewska@uniwersytetkaliski.edu.pl

Abstract: This study investigates the dietary patterns, health behaviors, and related determinants among young people in Poland, amid increasing lifestyle-related health concerns like obesity and poor nutrition. Understanding the factors influencing these behaviors is crucial for crafting effective public health strategies. The objective was to analyze young Poles' eating habits, their perceptions of health, and the role of various determinants in shaping these behaviors.
A survey was conducted among a representative sample of young Poles, gathering data on eating habits, health perceptions, and lifestyle choices. The survey focused on the respondents' understanding of healthy eating, sources of nutrition knowledge, and the influence of family, social media, and public health campaigns. Data were analyzed using descriptive statistics, and correlations between health behaviors and socio-demographic factors were examined.
The study revealed that 88% of young respondents prioritize health, with 73% acknowledging a link between nutrition and health. While most participants accurately described healthy eating and reported adherence to dietary guidelines, 43% engaged in emotional eating, which often led to compulsive behaviors associated with obesity. Social media, internet articles, and family were primary sources of nutrition knowledge. Despite their knowledge, fruit and vegetable consumption was below recommended levels, and physical activity levels were insufficient in nearly half of the respondents. Only 36% regularly read food labels, with many choosing products containing additives. Appearance-related stress was prevalent among 52% of respondents, while sleep deficits and lack of sufficient physical activity were common.
The findings suggest that while young Poles possess a high level of awareness regarding healthy eating, gaps remain in the application of this knowledge, particularly concerning emotional eating and inadequate fruit and vegetable consumption. Public health campaigns should be more effectively targeted to address these gaps and promote sustainable health behaviors from early childhood. Addressing emotional eating, enhancing physical activity, and improving nutrition education through effective channels like social media are key to improving public health outcomes.

Keywords: dietary habits; public health; young people; dietary rules; social media; social rules

Table S1. Survey

**Questions**	
Age	……………………………………….
Gender	……………………………………….
Height	……………………………………….
Weight	……………………………………….
Place of residence:	Village<br>Town/City
I am:	A primary school student<br>A secondary school student<br>A university student<br>I have completed my education – I am employed<br>Other ……………………………
How important is health to you?	Very important<br>Important<br>Indifferent<br>Not important
How would you rate the impact of healthy eating on health?	It has a major impact<br>It has a moderate impact<br>It has a minor impact<br>It has no impact
What do you think healthy eating means?	……………………………………………….
What has most influenced your knowledge about healthy eating? (Select up to 3 options)	Family<br>Friends/Peers<br>Educational programs in the media (guides, science programs, lifestyle shows, etc.)<br>Educational programs in school (e.g., “5 servings of fruits, vegetables, or juice”)<br>Social media<br>Internet articles<br>Public campaigns (e.g., "Don't serve yourself disease!" or “Planning for a long life”)<br>Other ……………
Did your parents pay attention to healthy eating?	Yes<br>No

	Sometimes
Is there a habit of cooking meals at home in your family?	Meals are regularly cooked at home<br>Meals are irregularly cooked at home, depending on my parents' or my schedule<br>Meals are rarely cooked at home<br>Meals are not cooked at home
Did your parents talk to you about what kind of diet is healthy?	Yes<br>No
What does food mean to you?	A means to sustain basic life functions<br>A form of reward<br>A way to boost my mood
Do you agree with the statement that regular meal times matter?	Yes<br>No
Do you eat breakfast every day?	Yes<br>No
Have you ever followed a diet or fast?	Yes<br>No
Why did you follow a diet or fast?	For health reasons
	To lose weight
	Due to illness
	Other reason (please specify) ……………
Is your appearance a source of stress for you?	Yes<br>No
Do you pay attention to the calorie content of your meals/snacks?	Yes<br>No<br>Sometimes
Do you think about how many calories you've consumed throughout the day?	Yes<br>No<br>Sometimes
Do you check the ingredients of the food products you buy?	Yes<br>No<br>Sometimes
	Yes

Do you try to avoid products containing harmful ingredients (preservatives, flavor enhancers, colorants, trans fats, etc.)?	No<br>Sometimes
Do you use apps to check product ingredients while shopping (e.g., Zdrowe Zakupy, Wiesz Co Jesz)?	Yes<br>No<br>Sometimes
How often do you consume highly processed foods? (ready-made meals, semi-processed foods, sweet and salty snacks, canned foods, sausages, soups, and sauces in powder form, etc.	Daily<br>Several times a week<br>Several times a month<br>Every 2-3 months<br>Less frequently than every 2-3 months<br>I never eat highly processed food
Is the higher price of healthier products a barrier to purchasing them?	Yes<br>No
Would you buy organic/healthier products if you didn't have to worry about the price?	Yes<br>No<br>I don't know
Do you follow your own dietary rules (eating at specific times, avoiding snacks, avoiding sugar, avoiding trans fats, quitting fast food, avoiding energy drinks, drinking a certain amount of water, etc.)?	Yes<br>No
What are your dietary rules?	……………………………………………………
Do you allow yourself exceptions to your dietary rules?	Yes, very often<br>Yes, sometimes<br>Very rarely<br>Never
Do you feel guilty thinking you are eating something unhealthy/too much/too late, etc.?	Yes<br>No
Are you satisfied with your health?	Yes<br>No
Are you satisfied with your appearance?	Yes<br>No

	I am mostly satisfied, but I would change certain aspects of my appearance
Do you ever overeat?	Yes<br>No<br>Sometimes
How would you describe your weight?	My weight is within the normal range<br>I am underweight<br>I weigh up to 5 kg more than I should<br>I weigh 5-10 kg more than I should<br>I weigh more than 10 kg above the normal range
Do you eat more in stressful situations?	Yes<br>No<br>Sometimes
Are you physically active/do you play sports?	I am physically active every day<br>I am physically active several times a week<br>I am physically active several times a month<br>My physical activity is sporadic<br>I am not physically active
Do well-known individuals (athletes, celebrities, actors, etc.) promoting a healthy lifestyle influence your dietary habits?	Yes (please provide an example of such person(s): …………………)<br>No
How often do you watch/read content related to healthy eating?	Often<br>Sometimes<br>Rarely<br>I only watch/read by accident<br>I do not watch/read healthy eating content
How often do you eat the following foods: [Table S1a]	
How often do you have meals? [Table S1b]	
Which of the following products do you snack on between meals, and how often? [Table S1c]	
	6 hours or less

How much time do you spend sleeping on weekdays?	7-8 hours<br>9 hours or more
How much time do you spend sleeping on weekends?	6 hours or less<br>7-8 hours<br>9 hours or more

Table S1a. How often do you eat the following foods?

**Product**	**Daily**	**Several Ti-mes a week**	**Once a week**	**Several Ti-mes a month**	**Once ever 2-3 months**	**Once every 6 months**	**Never**
**Fast food**							
**Salty snacks**							
**Sweets**							
**Sugary soft drinks**							
**Energy drinks**							
**Fruits**							
**Vegetables**							
**Grains**							
**Meat and cold cuts**							
**Dairy products**							
**Fish**							

**eggs**							

Table S1b. How often do you have meals?

**Product**	**Daily**	**Several Times a week**	**Once a week**	**Several Times a month**	**Once ever 2-3 months**	**Once every 6 months**	**Never**
First brecfast							
Second breakfast							
Lunch							
Afternoon snack							
Dinner							
Hot meal							
Snacking between meals							

Table S1c. Which of the following products do you snack on between meals, and how often?

Product	Daily	Several Times a week	Once a week	Several Times a month	Once ever 2-3 months	Once every 6 months	Never
Crackers, crisps							
Fries, hamburgers							
Sweets							
Fruits or vegetables							
Yogurts, dairy snacks							
Coca-Cola							
Other sugary soft drinks							
Energy drinks							

Table S2. Questions from the form

Questions		N=613	%
How important is health to you?	Very important	197	32
	Important	345	56
	Indifferent	65	11
	Not important	6	1
	It has a major impact	447	73

How would you rate the impact of healthy eating on health?	It has a moderate impact	148	24
	It has a minor impact	7	1
	It has no impact	11	2
Did your parents pay attention to healthy eating?	Yes	197	32
	No	125	21
	Sometimes	291	47
Is there a habit of cooking meals at home in your family?	Meals are regularly cooked at home	495	81
	Meals are irregularly cooked at home, depending on my parents' or my schedule	104	17
	Meals are rarely cooked at home	5	1
	Meals are not cooked at home	9	1
Did your parents talk to you about what kind of diet is healthy?	Yes	325	53
	No	288	47
Is your appearance a source of stress for you?	Yes	319	52
	No	294	48
How often do you consume highly processed foods? (ready-made meals, semi-processed foods, sweet and salty snacks, canned foods, sausages, soups, and sauces in powder form, etc.	Daily	57	9
	Several times a week	239	39
	Several times a month	234	38
	Every 2-3 months	40	7
	Less frequently than every 2-3 months	33	5
	I never eat highly processed food	10	2
Is the higher price of healthier products a barrier to purchasing them?	Yes	369	60
	No	244	40
Would you buy organic/healthier products if you didn't have to worry about the price?	Yes	441	72
	No	49	8
	I don't know	123	20
Do you follow your own dietary rules (eating at specific times, avoiding snacks, avoiding sugar, avoiding trans fats, quitting fast food, avoiding energy drinks, drinking a certain amount of water, etc.)?	Yes	319	52
	No	294	48
Do you allow yourself exceptions to your dietary rules?	Yes, very often	116	19
	Yes, sometimes	346	56
	Very rarely	126	21

	Never	25	4
Do you feel guilty thinking you are eating something unhealthy/too much/too late, etc.?	Yes	304	50
	No	309	50
Are you satisfied with your health?	Yes	375	61
	No	238	39
Are you satisfied with your appearance?	Yes	154	25
	No	145	24
	I am mostly satisfied, but I would change certain aspects of my appearance	314	51
Do you ever overeat?	Yes	186	30
	No	196	32
	Sometimes	231	38
How would you describe your weight?	My weight is within the normal range	319	52
	I am underweight	94	15
	I weigh up to 5 kg more than I should	85	14
	I weigh 5-10 kg more than I should	68	11
	I weigh more than 10 kg above the normal range	47	8
Do you eat more in stressful situations?	Yes	111	18
	No	341	56
	Sometimes	161	26
Are you physically active/do you play sports?	I am physically active every day	89	15
	I am physically active several times a week	210	34
	I am physically active several times a month	112	18
	My physical activity is sporadic	147	24
	I am not physically active	55	9
How often do you watch/read content related to healthy eating?	Often	108	18
	Sometimes	218	36
	Rarely	153	25
	I only watch/read by accident	86	13
	I do not watch/read healthy eating content	48	8
